# Comparison of CYP2C9 Activity in Ethiopian and Non-Ethiopian Jews Using Phenytoin as a Probe

**DOI:** 10.3389/fphar.2020.566842

**Published:** 2020-09-24

**Authors:** Zahi Abu Ghosh, Shoshana Alamia, Chanan Shaul, Yoseph Caraco

**Affiliations:** Clinical Pharmacology Unit, Division of Medicine, Hadassah-Hebrew University Medical Center, Jerusalem, Israel

**Keywords:** CYP2C9, cytochrome P450 complex subunit 2C9, phenytoin, *in vivo* activity, ethnicity, genetic polymorphism

## Abstract

The pharmacokinetics of CYP2C9 substrates is characterized by substantial interethnic variability. The objective of the study was to compare CYP2C9 activity by using Phenytoin Metabolic Ratio (PMR) between Ethiopian and non-Ethiopian Jews. PMR was derived from the ratio of p-HPPH in 24 h urine collection to plasma phenytoin, 12 h (PMR24/12) or 24 h (PMR24/24) after the administration of 300 mg phenytoin. Analysis of CYP2C9*2, *3, *5, *6, *8, and *11 was carried by direct sequencing. PMR was significantly correlated with *CYP2C9* genotype in both groups (p < 0.002). Mean PMR values were similar among Ethiopians and non-Ethiopians despite the fact that the fraction of non-carriers of *CYP2C9* variant alleles was significantly different (85 vs. 53%, respectively, p < 0.001). However, among non-carriers of CYP2C9*2, *3, *5, *6, *8, and *11 variant alleles, PMR24/12 and PMR24/24 values were 30 and 34% greater respectively in the non-Ethiopians group (p < 0.001). In conclusion—CYP2C9 activity as measured by PMR is similar in Ethiopian and non-Ethiopian Jews. However, among non-carriers of *CYP2C9* variant alleles accounting for 85% of Ethiopian Jews, CYP2C9 activity is decreased by approximately one third as compared with non-Ethiopian Jews. Unique genetic CYP2C9 polymorphisms occurring only in Ethiopians may account for this difference.

## Introduction

The metabolism of approximately 50% of commonly used drugs is mediated by three members of the cytochrome P450 superfamily of oxidative enzymes, CYP3A4, CYP2D6 and CYP2C9 (Zanger and Schwab, 2013; Daly et al., 2017). The latter accounts for 20% of hepatic P450 content and is the major catalytic enzyme responsible for the metabolism of about 15% of frequently prescribed drugs (Zanger and Schwab, 2013; Daly et al., 2017). The list of CYP2C9 substrates consists of several important drug and drug classes among them most NSAIDs (diclofenac, naproxen, lornoxicam, ibuprofen), coumarin anticoagulants [(*S*)-warfarin, phenprocoumon, acenocoumarol], angiotensin receptor antagonists (losartan, irbesartan), sulfonylurea antidiabetic drugs (glimepiride, glyburide, tolbutamide), and the anticonvulsant phenytoin (Zanger and Schwab, 2013; Daly et al., 2017).

CYP2C9 catalytic activity is characterized by marked interindividual variability. For example, the 8 h urine ratio of losartan to E-3174, a metabolite produced predominantly by CYP2C9, exhibited 40 folds variation among 39 healthy Caucasian subjects (Yasar et al., 2002). A significant fraction of this variability has been ascribed to genetic polymorphism in the gene encoding for CYP2C9. Indeed, *CYP2C9* gene is highly polymorphic and as of to date more than 60 variant alleles, some of which are associated with reduced function or even non-functional protein have been described (PharmGKB, 2020). Reduced clearance of the CYP2C9 substrates such as (*S*)-warfarin and phenytoin among carriers of defective alleles could results in significant bleeding complications or neurotoxicity respectively (Kidd et al., 2001; Jorgensen et al., 2012; Kawai et al., 2014; Calderon-Ospina et al., 2020).

CYP2C9 activity may be modulated by non-genetic factors such as gender, cigarettes smoking, food constituents, and age (Kimura et al., 2010; Dorado et al., 2012; Hatta et al., 2015). In addition, ethnicity is considered an important determinant explaining substantial difference in the pharmacokinetics of CYP2C9 substrates among subjects with different ethnic origin (Llerena et al., 2014; Hatta et al., 2015). It has been speculated that the observed marked variability in warfarin dose requirement between Caucasians and African Americans may be partly related to racial differences in the activity of CYP2C9, the predominant enzyme mediating the metabolism of (*S*)-warfarin (Perera et al., 2011; Perera et al., 2013; Hernandez et al., 2015). Findings obtained from extensive research imply that the differences can be categorized into 3 major classes, variability in the frequency of common allelic variants (i.e. CYP2C9*2 & CYP2C9*3), the existence of ethnic specific allelic variants present almost solely in patients of African ancestry (i.e. CYP2C9*5, CYP2C9*6, CYP2C9*8, & CYP2C9*11), and different consequences of identical polymorphisms presents in both Caucasians and Africans (i.e. rs12777823) (Perera et al., 2011; Perera et al., 2013). However, in sharp contrast to the abundance of genotypic data only a handful of studies have utilized phenotypic tools to evaluate the overall (genetic and non-genetic) differences in CYP2C9 activity between subjects of different ethnic origins (Llerena et al., 2014; Céspedes-Garro et al., 2015; Hatta et al., 2015).

The Ethiopian Jews community residing in Israel consists currently of some 150,000 inhabitants (Central Bureau of Statistics, 2019). They have arrived to Israel during the last 40 years in several immigration waves and have gradually assimilated into the Israeli society. This population has been the center of several extensive studies that have characterized its unique genetic structure but pharmacogenetics studies are scarce and in particular CYP2C9 has not been evaluated (Britzi et al., 2000; Luo et al., 2004; Ronen et al., 2010; Yang et al., 2014). The purpose of the current study was to compare *CYP2C9* genotype and activity as measured by using phenytoin as a probe drug between Ethiopian Jews and a control group consisting of non-Ethiopian Jews.

## Subjects and Methods

### Subjects

Three hundred healthy subjects, 150 of Jewish Ethiopian ancestry and 150 non-Ethiopian Jews were enrolled into the study. Mean (± SD) Phenytoin Metabolic Ratio (PMR) in our previous studies was 8.5 ± 4.5 ml/min. We used this information to calculate the size of the study population to enable detection of 20% difference in PMR between study groups with a power of 90%, 5% α (type I error) and 0.05 level of significance. Potential candidates were considered as “Ethiopians” if both parents and grandparents were born in Ethiopia prior to immigration to Israel. All participants in the study, had to be non-smokers, at the age range of 18–50 years old, and healthy based on medical statement and a through physical examination. The presence of any chronic disease and the regular consumption of drugs including oral contraceptives and alcohol were considered as exclusion criteria. No matching in demographic characteristics was made between the Ethiopian and the non-Ethiopian study groups.

### CYP2C9 Phenotyping and Genotyping

Following an 8 h fasting, subjects were administered at approximately 20:00 a single dose of 300 mg phenytoin (3 capsules of Epanutin, Pfizer Ltd, 100 mg each) together with 250 ml of water. Fasting was continued for 4 additional hours post phenytoin intake. Immediately prior to phenytoin intake the subjects were requested to empty their bladder and to start urine collection for the next 24 h at two equal intervals, 12 h each: 0–12 h and 12–24 h. The volume of each urine collection was measured and 20 ml aliquots were immediately stored at -20°C for the future analysis of 5-(4-hydroxyphenyl)-5-phenylhydantoin (p-HPPH). Two blood samples, 5 ml each were drawn 12 and 24 h following phenytoin dosing. Plasma was separated and stored at -20°C for the measurement of plasma phenytoin concentration. DNA was extracted from an additional blood sample (3 ml) and used for *CYP2C9* genetic analysis. The study protocol was approved by the Hadassah Institutional Review Board and prior to enrollment and following a detailed explanation all subjects signed an informed consent form.

### Analysis of Phenytoin and p-HPPH

Plasma concentration of phenytoin and urine concentration of 5-(4-hydroxyphenyl)-5-phenylhydantoin (p-HPPH) were measured by two separate high-performance liquid chromatography methods. The plasma method was performed as previously described, with some modifications (Sawchuk and Cartier, 1980). Thus, 1 ml of plasma sample was filtered through a ChenElut CE1003 column (Varian, Harbor City, CA, USA) and eluted 10 min later twice with 4 ml of tert-butyl-methyl-ether. Following evaporation, the residue was reconstituted in 200 μl of the mobile phase, and a 30 μl aliquot was chromatographed on a reversed-phase 30 cm C_18_ μBondapak™ (10 μm) column from Waters Assoc. (Milford, MA, USA). Following incubation with β-glucuronidase and liquid-liquid extraction, 0.5 ml of urine was passed through a solid phase extraction - Strata NH2 Device. The elute was chromatographed on a reversed-phase 30 cm C_18_ μBondapak™ (10 μm) column. The quantification limits for plasma phenytoin and urine p-HPPH were 0.625 and 1.25 μg/ml, respectively. The inter-assay coefficients of variation for plasma phenytoin and urine p-HPPH were 2.12–5.67 and 2.27–2.85%, respectively. The corresponding values for intra-assay coefficients of variation were 0.32–2.26 and, 1.27–5.15%, respectively. It should be noted that only the production of (S)-p-HPPH is mediated by the activity of CYP2C9 whereas CYP2C19 mediates the production of (R)-p-HPPH. However, as previously shown by us the formation clearance of (R)-p-HPPH is 30-fold lower as compared with the formation clearance of (S)-p-HPPH and the urinary excretion (R)-p-HPPH accounts for less than 5% of total urine p-HPPH. Thus, the in-vivo activity of CYP2C9 can be reliably derived from the molar ratio of urinary content of (total) p-HPPH excreted over 24 h to mid-interval phenytoin plasma concentration (i.e. PMR24/12) or to phenytoin plasma concentration 24 h after dosing (i.e. PMR24/24), normalized to the duration of urine collection, as published previously (Caraco et al., 2001).

### *CYP2C9* Genetic Analysis

Genomic DNA was extracted from peripheral leukocytes using traditional phenol-chlorophorm extraction procedure. Identification of CYP2C9*2, CYP2C9*3, CYP2C9*5, CYP2C9*6, CYP2C9*8, and CYP2C9*11 was performed through 3 separate direct sequencing procedures (**BGI group,** **Shenzhen,** **Guangdong, China**). Briefly, 3 DNA fragments spanning exons 3 (CYP2C9*2 & CYP2C9*8), 5 (CYP2C9*6), and 7 (CYP2C9*3, CYP2C9*5 & CYP2C9*11) were amplified using the primers listed in **Table 1**. The identification of rs12777823 C>A at the promoter region of the CYP2C cluster gene was performed by PCR followed by digestion with BsrBI (New England, BioLab). Subjects not carrying any of the tested *CYP2C9* variant alleles were defined as carriers of the wild-type CYP2C9*1/*1 genotype.

**Table 1:** Forward and reverse primers used for sequencing of Exon 3 (CYP2C9*2 & CYP2C9*8), Exon 5 (CYP2C9*6), and Exon 7 (CYP2C9*3, CYP2C9*5 & CYP2C9*11).

	Forward Primer	Reverse Primer
Exon 3	CACTGGCTGAAAGAGCTAACAGAG	GTGATATGGAGTAGGGTCACCCAC
Exon 5	CAGAGCTTGGTATATGGTATG	GTAAACACAGAACTAGTCAACAA
Exon7	ACCTTCATGATTCATATACCCC	GCCATACATATGAGTTATGCAC

### Data Analysis

Demographic details and the distribution of *CYP2C9* as well as of *rs12777823* genotypes are presented separately for each of the study groups in **Table 2**. Urinary excretion of p-HPPH over 24 h, PMR24/12 and PMR24/24 values were log transformed prior to statistical comparison. Comparison of data between carriers of different genotypes (i.e. *CYP2C9*, *rs12777823*) within each ethnic group was performed using ANOVA followed by unpaired *t*-test. Comparison of pharmacokinetic parameters between the Ethiopian and the non-Ethiopian group was performed using unpaired student’s *t*-test. Differences between the study groups in the frequency of *CYP2C9* genotypes were analyzed using Chi-Square test. The impact of demographics (i.e. age, weight, and gender) and *CYP2C9* genotype on Log_10_PMR was evaluated through univariate analysis using Pearson correlation test. Variables that correlated with Log_10_PMR with a p value of less than 0.1 were entered into multiple regression analysis model using the stepwise approach. Urinary excretion of p-HPPH and PMR values are presented as geometric mean with the respective 95% confidence interval throughout the entire paper. Whenever comparison was made either within or between study cohorts the geometric mean ratio is given with the respective 95% confidence interval. Statistical analysis was performed using the SPSS software package (IBM SPSS Statistics, version 23, Chicago IL, USA.) and p value of less than 0.05 was considered to denote statistical significance.

**Table 2:** Demographic details, *CYP2C9* and *rs12777823* genotypes and allele frequency in Ethiopian and non-Ethiopian Jews.

	Ethiopians (N = 150)	Non-Ethiopians (N = 150)	p Value
Age (years)	24.9 ± 5.4	24.9 ± 5.8	NS
Male/Female	66/84	65/85	NS
Weight (kg)	62.3 ± 12.3	69.1 ± 13.6	<0.001
*CYP2C9* Genotype			<0.001
*1/*1	128	80	<0.001
*1/*2	5	35	<0.001
*1/*3	2	26	<0.001
*1/*11	12	0	<0.001
*2/*2	0	1	NS
*2/*3	0	6	P < 0.05
*2/*11	3	0	NS
*3/*3	0	1	NS
*3/*8	0	1	NS
Allele Frequency			
*1	0.917	0.737	<0.001
*2	0.027	0.143	<0.001
*3	0.007	0.117	<0.001
*8	0	0.003	–
*11	0.05	0	<0.001
			
rs12777823			0.065
* GG*	106	121	0.06
* GA*	39	28	NS
* AA*	5	1	NS
Allele Frequency			
* G*	0.837	0.900	0.065
* A*	0.163	0.100	0.065

## Results

All 300 subjects, 150 Ethiopian and 150 non-Ethiopian Jews that were enrolled completed the study. Mean age and the proportion of male to female were similar, but the weight of the Ethiopian Jews was in average 11% lower (**Table 2**). The frequency of CYP2C9*2 and CYP2C9*3 alleles was markedly greater among the non-Ethiopian as compared with the Ethiopian Jews (14.3 vs. 2.7%, p < 0.001 and 11.7 vs. 0.7% p < 0.001, respectively). CYP2C9*5 and *6 variant alleles were not found in either study groups. CYP2C9*8 was found only in one non-Ethiopian subject but in none of the Ethiopian participants. However, CYP2C9*11 was identified in 15 subjects of the Ethiopian but in none of the non-Ethiopian Jews. Thus the fraction of subjects defined as carriers of the wild-type CYP2C9*1/*1 genotype (i.e. non-carriers of CYP2C9*2, *3, *5, *6, *8, and *11) was markedly higher among the Ethiopian as compared with the non-Ethiopian Jews (0.85 vs. 0.53, respectively, p < 0.001). The distribution of *CYP2C9* genotypes was in accordance with Hardy-Weinberg equilibrium in both Ethiopian and non-Ethiopians (Chi-Square, goodness to fit, p > 0.3 and p > 0.8 respectively).

The content of p-HPPH in 24 h urine collection, PMR24/12 and PMR24/24 varied significantly among carriers of different *CYP2C9* genotypes in both Ethiopians and non-Ethiopians (ANOVA, p < 0.003) (**Tables 3** **–** **5**). Thus, among Ethiopian Jews carriers of the CYP2C9*1/*2, CYP2C9*1/*3, CYP2C9*1/*11, and CYP2C9*2/*11 genotypes exhibited an average of 28, 16, 46, and 62% reduction in PMR24/12 as compared with carriers of the wild-type CYP2C9*1/*1 genotype (p = 0.06, NS, p < 0.001 and p < 0.002 respectively). The corresponding values for the reduction in PMR24/24 were 32, 36, 58, and 65% (p = 0.09, NS, p < 0.001 and p < 0.002, respectively). Similarly, among the non-Ethiopian Jews, PMR24/12 was reduced by an average of 41, 53, and 77% in carriers of the CYP2C9*1/*2, CYP2C9*1/*3, and CYP2C9*2/*3 genotypes as compared with carriers of CYP2C9*1/*1 genotype, respectively (p < 0.001). The corresponding average reduction in PMR24/24 values among the carriers of the same genotypes was 45, 62, and 82% respectively, p < 0.001). Furthermore, the single non-Ethiopian subject carrying the CYP2C9*3/*3 genotype had an extremely low PMR24/12 value of 0.63 ml/min and PMR24/24 value of 0.57 ml/min.

**Table 3:** Urinary excretion of p-HPPH over 24 h according to *CYP2C9* and *rs12777823* genotype in the Ethiopian and non-Ethiopian groups.

	Ethiopian Jews		Non-Ethiopian Jews		Ethiopians/non-Ethiopians
	GM (µmol) (95% CI)	GMR (95% CI)	GM (µmol) (95% CI)	GMR (95% CI)	GMR (95% CI)
*CYP2C9*					
*1/*1	140 (131–149)	1	176 (164–190)	1	0.79 (0.72–0.88)
*1/*2	118 (99–142)	0.85 (0.61–1.18)	135 (124–147)	0.76 (0.67–0.87)	0.88 (0.69–1.11)
*1/*3	103 (61–174)	0.74 (0.44–1.24)	109 (98–122)	0.62 (0.54–0.71)	0.94 (0.63–1.41)
*1/*11	99 (83–118)	0.71 (0.57–0.88)	–	–	–
*2/*2	–	–	54	0.30 (0.16–0.59)	–
*2/*3	–	–	53 (43–64)	0.30 (0.23–0.39)	–
*2/*11	62 (42–92)	0.45 (0.29–0.68)	–	–	–
*3/*3	–	–	12	0.07 (0.04–0.13)	–
*3/*8	–	–	58	0.33 (0.17–0.65)	–
p Value	<0.001		<0.003		
*rs12777823*					
* GG*	139 (129–149)	1	135 (124–147)	1	1.03 (0.92–1.15)
* GA*	124 (111–140)	0.89 (0.78–1.03)	167 (140–199)	1.24 (1.02–1.50)	0.74 (0.61–0.91)
* AA*	81 (60–109)	0.58 (0.41–0.81)	126	0.93 (0.37–2.34)	0.64 (0.31–1.34)
p Value	<0.003		0.091		

(GM, Geometric Mean; GMR, Geometric Mean Ratio).

**Table 4:** Phenytoin Metabolic Ratio 24/12 according to *CYP2C9* and *rs12777823* genotype in the Ethiopian and non-Ethiopian groups.

	Ethiopian Jews		Non-Ethiopian Jews		Ethiopians/non-Ethiopians
	GM (ml/min) (95% CI)	GMR (95% CI)	GM (ml/min) (95% CI)	GMR (95% CI)	GMR (95% CI)
*CYP2C9*					
*1/*1	6.25 (5.70–6.86)	–	8.94 (8.11–9.86)	–	0.70 (0.61–0.80)
*1/*2	4.51 (3.18–6.41)	0.72 (0.45–1.16)	5.30 (4.66–6.04)	0.59 (0.50–0.70)	0.85 (0.60–1.22)
*1/*3	5.23 (1.48–18.41)	0.84 (0.40–1.75)	4.18 (3.61–4.84)	0.47 (0.39–0.56)	1.25 (0.73–2.14)
*1/*11	3.37 (2.66–4.27)	0.54 (0.40–0.73)	–	–	
*2/*2			2.26	0.25 (0.11–0.61)	–
*2/*3			2.08 (1.83–2.37)	0.23 (0.16–0.33)	–
*2/*11	2.39 (1.53–3.71)	0.38 (0.21–0.70)	–		–
*3/*3	–		0.63	0.07 (0.03–0.17)	–
*3/*8	–		2.08	0.23 (0.10–0.56)	–
p Value	<0.001		<0.002		–
*rs12777823*					
* GG*	6.22 (5.62–6.89)	–	6.06 (5.44–6.75)	–	1.03 (0.89–1.19)
* GA*	5.10 (4.34–6.00)	0.82 (0.68–0.99)	7.54 (5.99–9.49)	1.24 (0.97–1.59)	0.68 (0.52–0.88)
* AA*	2.93 (1.38–6.22)	0.47 (0.29–0.76)	5.99	0.99 (0.30–3.25)	0.49 (0.08–3.10)
p Value	<0.002		0.221		

(GM, Geometric Mean; GMR, Geometric Mean Ratio).

**Table 5:** Phenytoin Metabolic Ratio 24/24 according to *CYP2C9* and *rs12777823* genotype in the Ethiopian and non-Ethiopian groups.

	Ethiopian Jews		Non-Ethiopian Jews		Ethiopians/non-Ethiopians
	GM (ml/min) (95% CI)	GMR (95% CI)	GM (ml/min) (95% CI)	GMR (95% CI)	GMR (95% CI)
* CYP2C9*					
*1/*1	9.36 (8.48–10.33)	–	14.28 (12.59–16.21)	–	0.66 (0.56–0.77)
*1/*2	6.40 (3.95–10.36)	0.68 (0.41–1.13)	7.80 (6.75–9.01)	0.55 (0.44–0.67)	0.82 (0.55–1.23)
*1/*3	5.95 (1.04–34.12)	0.64 (0.29–1.40)	5.48 (4.71–6.38)	0.38(0.30–0.49)	1.09 (0.62–1.90)
*1/*11	3.90 (3.08–4.93)	0.42 (0.30–0.58)	–	–	–
*2/*2	–	–	2.56	0.18 (0.06–0.55)	–
*2/*3	–	–	2.54 (1.89–3.40)	0.18 (0.11–0.28)	–
*2/*11	3.31 (1.44–7.62)	0.35 (0.19–0.68)	–	–	–
*3/*3	–	–	0.57	0.04 (0.01–0.13)	–
*3/*8	–	–	3.37	0.24 (0.08–0.73)	–
p Value	<0.001		<0.001		
*rs12777823*					
* GG*	9.57 (8.59–10.68)	–	8.95 (7.84–10.20)	–	1.07 (0.90–1.27)
* GA*	6.59 (5.53–7.86)	0.69 (0.56–0.85)	11.32 (8.56–14.97)	1.27 (0.94–1.71)	0.58 (0.43–0.79)
* AA*	3.34 (1.73–6.43)	0.35 (0.21–0.58)	12.14	1.36 (0.32–5.76)	0.28 (0.06–1.37)
p Value	<0.001		0.288		

(GM, Geometric Mean; GMR, Geometric Mean Ratio).

**Figure 1:**
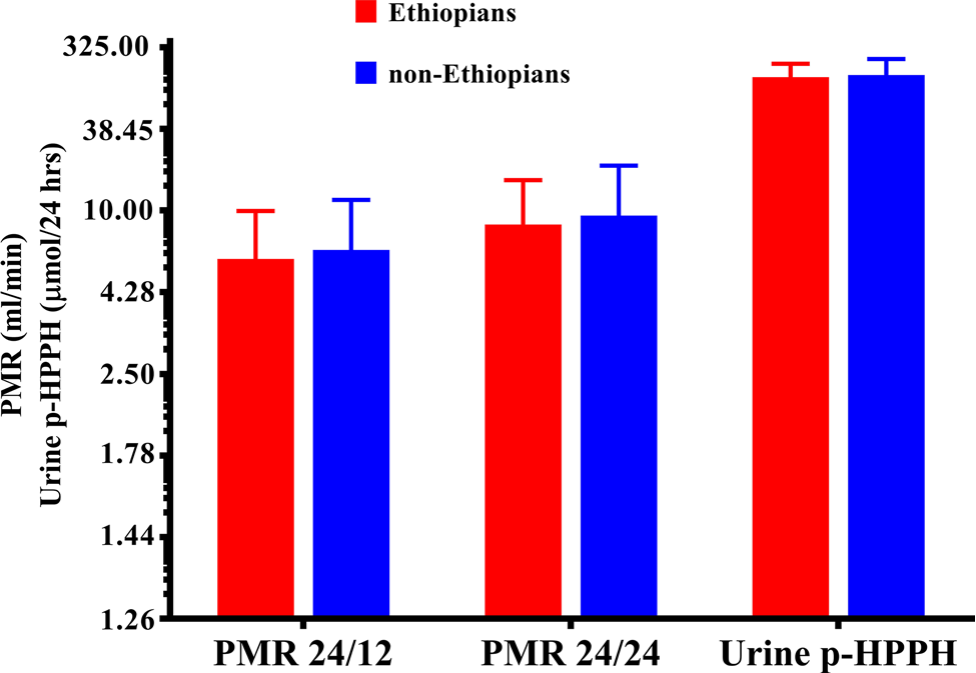
The molar amount of p-HPPH in 24 urine collection, PMR24/12 and PMR24/24 in Ethiopian (red bars) and non-Ethiopian (blue bars) Jews. Log_10_ transformed data was used for comparison with anti log_10_ values presented on the y-axis. (Statistical comparison between Ethiopian and non-Ethiopian groups was performed by *t*-test on log_10_ transformed data).

**Figure 2:**
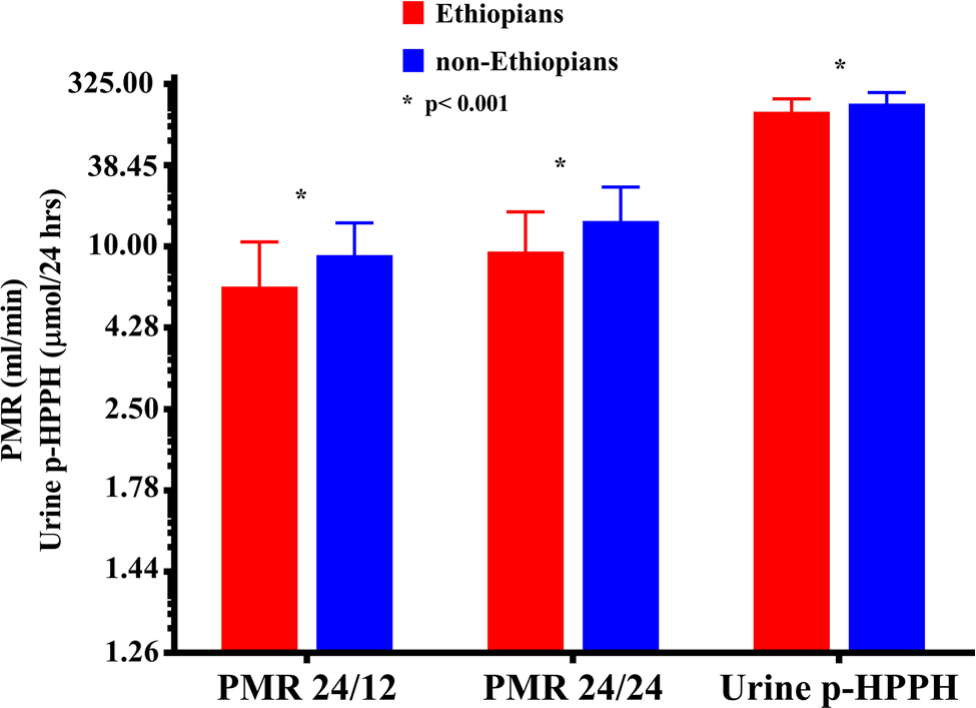
The molar amount of p-HPPH in 24 urine collection, PMR24/12 and PMR24/24 among non-carriers of CYP2C9*2, *3, *5, *6, *8, and *11 Ethiopian (red bars) and non-Ethiopian (blue bars) Jews. Log_10_ transformed data was used for comparison with anti log_10_ values presented on the y-axis. (Statistical comparison between Ethiopian and non-Ethiopian groups was performed by *t*-test on log_10_ transformed data).

Urinary excretion of p-HPPH over 24 h, PMR24/12 and PMR24/24 were numerically greater in the non-Ethiopian group by an average of 6, 9, and 11% respectively but none of these differences reached statistical significance (**Figure 1**). However, when only carriers of CYP2C9*1/*1 genotype were considered, urinary excretion of p-HPPH, PMR24/12, and PMR24/24 were significantly greater in the non-Ethiopian Jews by an average of 21, 30, and 34% respectively, p < 0.001 (**Figure 2**).

**Figure 3:**
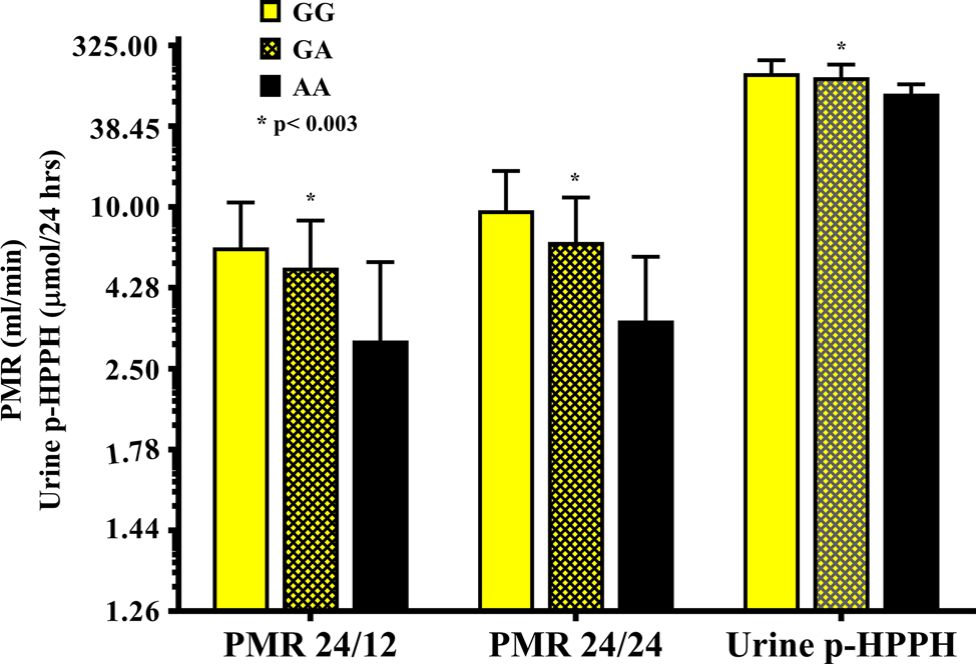
The molar amount of p-HPPH in 24 urine collection, PMR24/12 and PMR24/24 in Ethiopian carriers of *rs12777823 GG* (yellow bars), *GA* (yellow hatched bars), and *AA* (black bars) genotypes. Log_10_ transformed data was used for comparison with anti log_10_ values presented on the y-axis. (Statistical comparison between carriers of different *rs12777823* genotypes was performed by ANOVA on log_10_ transformed data).

**Figure 4:**
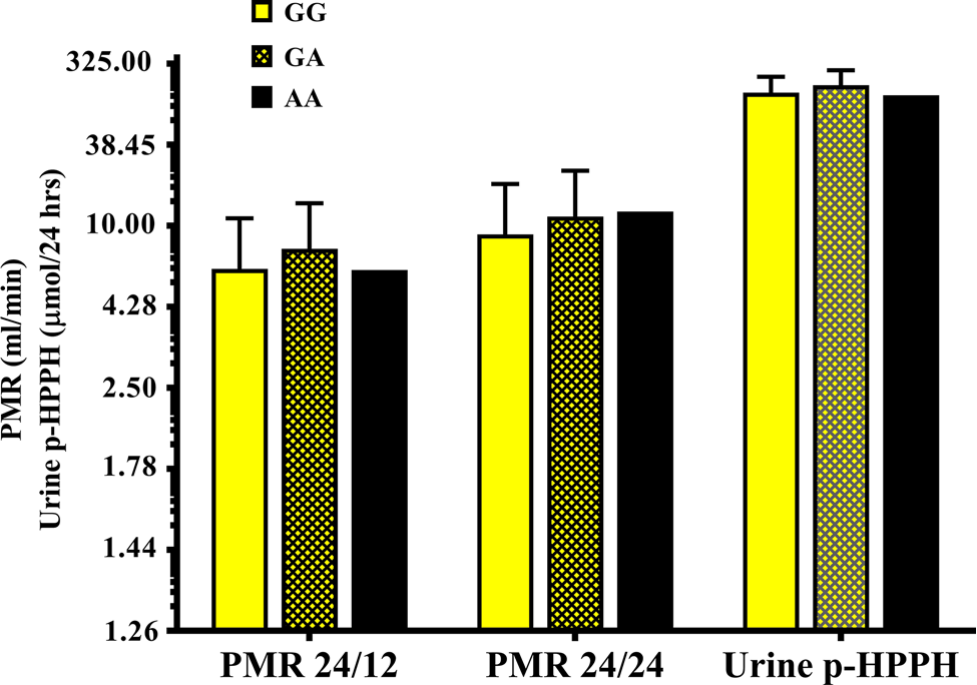
The molar amount of p-HPPH in 24 urine collection, PMR24/12 and PMR24/24 in non-Ethiopian carriers of *rs12777823* *GG* (yellow bars), *GA* (yellow hatched bars), and *AA* (black bars) genotypes. Log_10_ transformed data was used for comparison with anti log_10_ values presented on the y-axis. (Statistical comparison between carriers of different *rs12777823* genotypes was performed by ANOVA on log_10_ transformed data).

The distribution of *rs12777823* genotypes is described in **Table 2**. The number of subjects carrying at least a single A allele was numerically higher among the Ethiopian as compared with the non-Ethiopian group (44 vs. 29, respectively, p = 0.06). In the Ethiopian Jews *rs12777823* genotype was significantly associated with the urinary excretion of p-HPPH over 24 h, PMR24/12 and PMR24/24 (p < 0.003, p < 0.002, and p < 0.001, respectively) (**Tables 3** **–** **5**, **Figure 3**). Thus, urinary excretion of p-HPPH was numerically lower by 11% among carriers of the GA genotype and significantly lower by 42% among carriers of the AA genotype as compared with carriers of the GG genotype (NS and p < 0.002, respectively). Similarly, PMR24/12 was significantly lower by an average of 18 and 53% among carriers of the GA and the AA genotype as compared with carriers of the GG genotype (p < 0.05 and p < 0.003, respectively). The corresponding decrease in PMR24/24 values among carriers of GA and AA genotypes as compared with carriers of GG genotype was 31 and 65%, p < 0.001, respectively. An opposite trend was noted among the non-Ethiopian Jews (**Tables 3** **–** **5**, **Figure 4**). Thus, the excretion of p-HPPH was in average 24% higher among carriers of the GA as compared with carriers of the GG genotype (p < 0.03). In addition, PMR24/12 and PMR24/24 were numerically higher by an average of 24 and 27% in carriers of GA as compared to carriers of the GG genotype respectively, but these differences did not reach statistical significance. Among carriers of the CYP2C9*1/*1 genotype, the association noted in the Ethiopian group between rs12777823 genotype and urinary excretion of p-HPPH, PMR24/12, and PMR24/24 was significantly attenuated (**Table 6**).

**Table 6:** Urinary excretion of p-HPPH over 24 h, PMR24/12 and PMR24/24 according to *rs12777823* genotype among non-carriers of CYP2C9*2, *3, *5, *6, *8, and *11 in the Ethiopian and non-Ethiopian groups.

	Ethiopian Jews		Non-Ethiopian Jews		Ethiopians/non-Ethiopians
	p-HPPH		p-HPPH		
	GM (µmol) (95% CI)	GMR (95% CI)	GM (µmol) (95% CI)	GMR (95% CI)	GMR (95% CI)
* GG*	141 (130–152)	1	172 (158–187)	1	0.82 (0.73–0.92)
*GA*	142 (126–161)	1.01 (0.86–1.19)	193 (164–226)	1.12 0.95–1.32)	0.74 (0.61–0.90)
*AA*	82 (40–172)	0.59 (0.34–1.01)	126	0.73 (0.38–1.40)	0.66 (0.18–2.34)
p Value	p = 0.126		p = 0.233		
	**PMR24/12**		**PMR24/12**		
	**GM (ml/min)** **(95% CI)**	**GMR** **(95% CI)**	**GM (ml/min)** **(95% CI)**	**GMR** **(95% CI)**	**GMR** **(95% CI)**
*GG*	6.34 (5.70–7.06)	1	8.96 (8.00–10.03)	1	0.71 (0.60–0.83)
*GA*	6.12 (5.07–7.37)	0.96 (0.77–1.21)	9.07 (7.31–11.26)	1.01 (0.81–1.26)	0.67 (0.51–0.89)
*AA*	4.23	0.67 (0.31–1.44)	5.99	0.67 (0.28–1.58)	0.71
p Value	p = 0.547		p = 0.657		
	**PMR24/12**		**PMR24/12**		
	**GM (ml/min)** **(95% CI)**	**GMR** **(95% CI)**	**GM (ml/min)** **(95% CI)**	**GMR** **(95% CI)**	**GMR** **(95% CI)**
*GG*	9.85 (8.80–11.02)	1	14.27 (12.26–16.61)	1	0.69 (0.57–0.83)
*GA*	8.17 (6.72–9.93)	0.83 (0.65–1.05)	14.42 (11.14–18.68)	1.011 (0.76–1.35)	0.57 (0.42–0.77)
*AA*	4.33	0.44 (0.20–0.99)	12.14	0.85 (0.27–2.68)	0.36
p Value	p < 0.05		p = 0.957		

(GM, Geometric Mean; GMR, Geometric Mean Ratio).

All variable that were correlated with Log_10_PMR at a significant level of p < 0.1 were entered into a multiple regression model using the stepwise approach. The final model consisted of CYP2C9*2, CYP2C9*3 and weight in both study groups but the presence of CYP2C9*11 was included only in the Ethiopian Jews model for both Log_10_PMR24/12 and Log_10_PMR24/24 (**Tables 7** and **8**, respectively). In addition, among Ethiopians the intronic variant *rs9332121* T>A and *rs12777823* G>A were also included in the regression model of PMR24/12 and PMR24/24, respectively. The regression model could explain 64 and 63% of the variability in Log_10_PMR24/12 and Log_10_PMR24/24 in non-Ethiopian but only 39 and 45% in Ethiopians, respectively. The same variables in addition to ethnicity were used to construct a multiple regression model for the combined cohort consisting of both Ethiopian and non-Ethiopian Jews. The model could predict 52 and 56% of the variability in Log_10_PMR24/12 and Log_10_PMR24/24 respectively, with ethnic origin accounting for approximately 3%.

**Table 7:** Multiple regression model for the prediction of Log_10_PMR24/12 among Ethiopian Jews, non-Ethiopian Jews, and the entire cohort of both Ethiopian and non-Ethiopian Jews.

	Ethiopian Jews N = 150		Non-Ethiopian Jews N = 150		Entire Cohort N = 300	
	% Variability	p Value	% Variability	p Value	% Variability	p Value
CYP2C9*2	3.1%	0.01	19.4%	0.001	8.3%	0.001
CYP2C9*3	2.0%	0.04	34.8%	0.001	17.5%	0.001
CYP2C9*11	13.2%	0.001	–	–	6.8%	0.001
*rs12777823G>A*	–	–	–	–	–	–
*rs9332121T>A*	2.8%	0.01	–	–	1.4%	0.004
Weight	17.8%	0.001	9.3%	0.001	15.0%	0.001
Ethnicity	NA	NA	NA	NA	2.9%	0.001
Total	38.9%	0.001	63.5%	0.001	51.9%	0.001

**Table 8:** Multiple regression model for the prediction of Log_10_PMR24/24 among Ethiopian Jews, non-Ethiopian Jews, and the entire cohort of both Ethiopian and non-Ethiopian Jews.

	Ethiopian Jews N = 150		Non-Ethiopian Jews N = 150		Entire Cohort N = 300	
	% Variability	p Value	% Variability	p Value	% Variability	p Value
CYP2C9*2	3.0%	0.006	17.5%	0.001	7.5%	0.001
CYP2C9*3	3.5%	0.004	35.7%	0.001	16.4%	0.001
CYP2C9*11	19.8%	0.001	–	–	9.3%	0.001
*rs12777823G>A*	3.4%	0.005	–	–	–	–
*rs9332121T>A*	–	–	–	–	1.7%	0.001
Weight	15.6%	0.001	9.3%	0.001	18.3%	0.001
Ethnicity	NA	NA	NA	NA	3.1%	0.001
Total	45.3%	0.001	62.5%	0.001	56.3%	0.001

## Discussion

Populations of different ethnic background may exhibit marked variability in response to commonly used drugs. A fraction of this variability is attributed to differences in the rate of drug metabolism. For example, out of the 167 new molecular entities (NMEs) approved by the FDA (2008–2013), the labelling of 35 included some ethnically based differences and racial variability in pharmacokinetics were included in the labeling of 19 approved drugs (Ramamoorthy et al., 2015; Bonham et al., 2016). The findings in the present study indicate that among carriers of the wild-type CYP2C9*1/*1 genotype the activity of this isoform is increased by an average range of 30–34% in non-Ethiopian as compared with Ethiopian Jews residing in Israel. Furthermore, similar trend towards lower average CYP2C9 activity was noted in Ethiopian Jews when the entire cohorts were compared, despite the fact that a significantly larger portion of the non-Ethiopian subjects carried at least one variant *CYP2C9* allele.

In the last 40 years, almost 90,000 Ethiopian Jews have arrived to Israel through several waves of immigration. Currently, the Israeli Ethiopian Jews community numbers approximately 150,000 residents accounting for about 1.7% of the Israeli population (Central Bureau of Statistics, 2019). However, until recently the metabolic profile of the Ethiopian Jews community was only scarcely assessed (Britzi et al., 2000; Luo et al., 2004). The reduced CYP2C9 activity among Ethiopian Jews noted in the present study in particular among carriers of the CYP2C9*1/*1 genotype may have significant clinical implications. As CYP2C9 mediates the metabolism of several drugs that are characterized by a narrow therapeutic window, extra caution should be exercised when such a drug like phenytoin is administered to a member of this community. Furthermore, Ethiopian Jews may be more susceptible to experience gastrointestinal bleeding complications during treatment with NSAIDs that are typical substrates of CYP2C9 (Pilotto et al., 2007; Daly et al., 2017). On the other hand, the blood lowering effect of losartan may be attenuated among Ethiopian Jews (Lajer et al., 2007; Daly et al., 2017).

The low frequency of CYP2C9*2 and CYP2C9*3 noted in our Ethiopian as compared with the non-Ethiopian cohort is in line with findings obtained in African populations including Ethiopians (Scordo et al., 2001). On the other hand, the frequency of CYP2C9*11 among Ethiopian Jews was 5% and higher than the frequency noted in other African population such as Ghanaians (2%) and Beninese (2.7%) (Allabi et al., 2003; Kudzi et al., 2009). Several studies have evaluated the activity of CYP2C9 in diverse populations using different probe drugs. The findings in many of these studies were helpful in defining the functional importance of *CYP2C9* allelic variants and thus explain variability in the pharmacokinetics of CYP2C9 substrates such as phenytoin, diclofenac, flurbiprofen, and losartan (Caraco et al., 2001; Yasar et al., 2002; Dorado et al., 2003; Allabi et al., 2004; Allabi et al., 2005; Vogl et al., 2015). Only handful of studies actually compared the activity between populations of different ethnic ancestry. In one such study, the ratio of diclofenac to 4-hydroxydiclofenac in 8 h urine collection, a marker of CYP2C9 activity, varied significantly across 3 different Hispanic populations, 2 from Cuba and one from Spain (Llerena et al., 2014). Specifically, Cubans of Caucasian origin exhibited an average 13% lower diclofenac metabolic ratio (i.e. higher CYP2C9 activity), as compared with Cuban with mixed Caucasian and African origin (Mestizos), a difference that persisted even when the comparison was limited to carriers of CYP2C9*1/*1 genotype. In another study, CYP2C9 activity was compared between Swedes and Koreans using losartan as a probe drug (Hatta et al., 2015). Losartan metabolic ratio (i.e. losartan to E-3174 in 8 h urine collection) was significantly higher in Swedes as compared with Korean and this difference remained valid when comparison was made between carriers of matched *CYP2C9* genotypes. However, no significant difference was noted in losartan metabolic ratio between 22 Swedes and 17 Spanish subjects (Yasar et al., 2002). In an extension of this study the authors have evaluated CYP2C9 activity using losartan MR in a small cohort consisting of 19 Beninese (Allabi et al., 2004). Among carriers of CYP2C9*1/*1 genotype, losartan MR was in the same range as the value noted in Spanish subjects carrying the same genotype and the authors suggested that the enzymatic activity of protein encoded by CYP2C9*1/*1 is most likely similar in Caucasian and Black Africans. The findings in the present study are at conflict with this conclusion. One possible explanation for the disparity is the fact that African populations should not be referred to as a homogenous entity and thus Beninese may genuinely differ from Ethiopians (Scordo et al., 2001; Allabi et al., 2003; Rotimi et al., 2017). On the other hand, the findings in the previous study were based on a very small groups (9 Beninese vs. 8 Spanish subjects) and therefore a valid comparison could not be made. Finally, based on population pharmacokinetic analysis, median clearance of (*S*)-warfarin, a prototype substrate of CYP2C9, was reduced by an average of 30% among African Americans as compared with Caucasians (Kubo et al., 2017; Ohara et al., 2019). This difference which was evident also for the comparison between carriers of CYP2C9*1/*1 genotype, corresponds to the 30–34% reduction in CYP2C9 activity noted in the present study in the Ethiopian Jews.

The reason for the difference in CYP2C9 activity between Ethiopian and non-Ethiopian Jews among carriers of allegedly the same CYP2C9*1/*1 genotype is currently unknown. Potential explanations may include but are not limited to unidentified genetic or epigenetic factors, environmental influences such as dietary habits and differences in host characteristics. The study groups were well balanced in terms of age and male to female ratio but the weight of the non-Ethiopian group was in average about 7 kg greater as compared with the Ethiopian Jews (69.1 ± 13.6 vs. 62.3 ± 12.3 kg, respectively). Body weight has been shown to correlate with (*S*)-warfarin clearance and theoretically the reduced CYP2C9 activity among the Ethiopian Jews could be at least in part attributed to average lower body weight (Lane et al., 2012). However, based on data from a previous publication, 7 kg difference in weight might be expected to result in a subtle 3.5% difference in (*S*)-warfarin clearance and thus could not account for the substantially greater difference in CYP2C9 activity noted in the present study. Furthermore, detailed pharmacokinetic studies with phenytoin failed to identify a significant difference in total body clearance among obese as compared with normal body weight subjects (Abernethy and Greenblatt, 1985).

The inhibitory effect of fruit juice as well as multiple spices and herbal substances on the enzymatic activity of cytochrome P450 isoforms including CYP2C9 is well documented (Hidaka et al., 2008; Kimura et al., 2010; Wang et al., 2020). The possibility that regular consumption of spices or herbs which are part of the traditional Ethiopian diet might inhibit the activity of CYP2C9 cannot be ruled out.

The difference in CYP2C9 activity was most apparent in the current study among carriers of the CYP2C9*1/*1 genotype. However, the definition of this genotype was per exclusion based on the absence of any of the 6 tested *CYP2C9* variant alleles. However, African populations are characterized by prominent genetic diversity which is often attributed to multiple rare genetic polymorphisms. Thus rare unidentified allelic variants which may encode for defective protein may exist in the Ethiopian Jews. Indeed, in the course of the genetic analysis we have identified two novel non-synonymous genetic polymorphisms occurring each in a single Ethiopian subject. One was a T to G substitution at position 10:94942237 and resulting in replacement of phenylalanine by cysteine at amino acid 126 (F126C) and the second one was a C to A at position 10:94981230 resulting in replacement of Proline by Threonine at amino acid 337 (P337T). Both genetic polymorphisms were evaluated as damaging with an extremely low SIFT (sorting intolerance from tolerance) score (Sim et al., 2012). Furthermore, the P337T genetic polymorphism designated as CYP2C9*58 has previously been reported in a warfarin-hypersensitive Chinese patient and when expressed in insect cells microsomes it exhibited decreased intrinsic clearance of losartan similar to that noted for CYP2C9*3 (Luo et al., 2014). Both novel genetic polymorphisms were detected in subjects that were assigned wrongly so as carriers of CYP2C9*1/*1 genotype and were associated with markedly reduced PMR values. The fact that the model could explain significantly less of the total variability in PMR among the Ethiopian as compared with the non-Ethiopian Jews is in line with the possibility that additional as yet unidentified possibly rare genetic polymorphism may exist in the Ethiopian group.

Two genetic polymorphisms in boundary of exon 3 were detected only in the Ethiopian groups. One was T>A at position 10:94942182 (*rs9332121*) found in 10 subjects, 8 carriers of CYP2C9*1/*1 genotype and 2 subjects carrying the CYP2C9*1/*11 genotype (Matimba et al., 2009). This polymorphism was associated with reduced PMR values and was included in the final regression model accounting for 2.8% of the variability in PMR24/12 among Ethiopians. The second polymorphism was a novel G>C at position 10:94942187 occurring in a single Ethiopian subject carrying CYP2C9*1/*1 genotype displaying markedly low PMR values. These intronic genetic variants identified only in Ethiopian subjects were located nearby or at the splice site suggesting that they may be functionally important.

Among the Ethiopian group carriage of rs12777823 A allele was associated with significant decrease in CYP2C9 activity as evaluated by PMR thus corroborating previous findings which were based on (*S*)-warfarin clearance (Perera et al., 2013). However, in the non-Ethiopian group, a trend towards higher activity of CYP2C9 among carriers of the rs12777823 A allele was noted. Thus, when CYP2C9 activity was compared between the entire Ethiopian and non-Ethiopian cohorts, the attenuating effect of rs12777823 genetic polymorphism among the Ethiopian Jews might have cancel out the impact of CYP2C9*2 and *3 which were markedly more abandoned in the non-Ethiopian group. The opposing effect of rs12777823 genetic polymorphism on PMR among Ethiopians vs. non-Ethiopians was less apparent when comparison was made only among carriers of CYP2C9*1/*1 genotype. Thus the difference in CYP2C9 activity noted between Ethiopian and non-Ethiopian Jews carrying the CYP2C9*1/*1 genotype cannot be accounted for by this genetic polymorphism.

Our study has certain limitations. Enrollment into the study was not based on matching of subjects characteristics between the Ethiopian and the non-Ethiopian groups. Although age and gender were similar, there was a significant difference in average weight with Ethiopian Jews weighting approximately 7 kg less. Thus we cannot rule out the possibility that other demographic details or personal habits (i.e. diet) that might influence CYP2C9 activity varied between the study groups. However, the study was planned on purpose to compare CYP2C9 activity between these communities under “real-life” conditions representing the average Ethiopian and non-Ethiopian subjects residing in Israel. The size of the study group was large enough to allow adequate representation of both communities. The PMR procedure involves urine collection over a period of 24 h at two intervals, and as such it is heavily dependent on subject’s compliance. Previous studies among kidney stone formers have indicated that roughly only half of the urine collections were judged as accurate (Boyd et al., 2018). Theoretically, the reduced PMR values among Ethiopian as compared with the non-Ethiopian Jews could reflect reduced accuracy in urine collection among the former group. However, urine volumes did not vary between the Ethiopian and the non-Ethiopian groups (0–12 h: 844 ± 522 vs. 870 ± 514 ml, respectively, p > 0.5; 12–24 h: 1,096 ± 679 vs. 1,082 ± 611 ml, respectively, p > 0.5). Thus the possibility of bias introduction due to difference in compliance is less likely.

In conclusion: Among carriers of CYP2C9*1/*1 genotype, PMR, a marker of CYP2C9 activity is reduced in Ethiopian as compared with non-Ethiopian Jews residing in Israel. Carriers of this genotype accounting for 85% of Ethiopian Jews enrolled into the study, may be at increased risk to experience adverse drug effects when treated by CYP2C9 substrate characterized by a narrow therapeutic window such as phenytoin or when administered common doses of NSAIDs. The mechanism for the reduced activity of CYP2C9 activity among Ethiopian Jews could be related to environmental factors (i.e. dietary habits), epigenetic factors or the presence of unique ethnically specific *CYP2C9* allelic variants encoding for malfunctioned protein.

## Data Availability Statement

The raw data supporting the conclusions of this article will be made available by the authors, without undue reservation, to any qualified researcher.

## Ethics Statement

The studies involving human participants were reviewed and approved by Hadassah Institutional Review Board. The participants provided their written informed consent to participate in this study.

## Author Contributions

ZA—Helped in designing the research, performed the research, analyzed the data, and took part in writing the manuscript. SA—Performed the research and helped in data analysis. CS—Helped in designing the study, performed the research, helped in data analysis, and writing of the manuscript. YC—Designed the study, analyzed the data, and drafted the paper.

## Conflict of Interest

The authors declare that the research was conducted in the absence of any commercial or financial relationships that could be construed as a potential conflict of interest.
